# A Review of the Occurrence of Metals and Xenobiotics in European Hedgehogs (*Erinaceus europaeus*)

**DOI:** 10.3390/ani14020232

**Published:** 2024-01-11

**Authors:** Sophie Lund Rasmussen, Cino Pertoldi, Peter Roslev, Katrin Vorkamp, Jeppe Lund Nielsen

**Affiliations:** 1 Wildlife Conservation Research Unit, The Recanati-Kaplan Centre, Department of Biology, University of Oxford, Tubney House, Tubney, Abingdon OX13 5QL, UK; 2 Department of Chemistry and Bioscience, Aalborg University, 9220 Aalborg, Denmark; cp@bio.aau.dk (C.P.); pr@bio.aau.dk (P.R.); jln@bio.aau.dk (J.L.N.); 3 Linacre College, University of Oxford, St. Cross Road, Oxford OX1 3JA, UK; 4 Aalborg Zoo, 9000 Aalborg, Denmark; 5 Department of Environmental Science, Aarhus University, 4000 Roskilde, Denmark; kvo@envs.au.dk

**Keywords:** European hedgehogs, *Erinaceus europaeus*, xenobiotics, heavy metals, environmental pollution, toxicants, target screening, non-target screening, bioaccumulation, wildlife conservation

## Abstract

**Simple Summary:**

The European hedgehog (*Erinaceus europaeus*) is a popular visitor in gardens and recreational areas all over Europe, but hedgehog populations are declining. Research exploring the causes of the decline, including exposure to potentially harmful pollutants and metals, may provide relevant information to improve conservation initiatives to protect this species in the wild. Hedgehogs are ground-dwelling mammals, feeding on a range of different food items such as insects, slugs, snails, and earthworms but also eggs, live vertebrates, and carrion, and therefore come into close contact with pollutants present in their habitats and in their prey. This review investigated published research on the occurrence of metals and pollutants in hedgehogs and found that a vast range of different pesticides; rodenticides; persistent organic pollutants (POPs), including organochlorine compounds and brominated flame retardants (BFRs); as well as toxic heavy metals could be detected in samples from hedgehogs representing different European countries. Due to their ecology, combined with the opportunity to apply non-invasive sampling techniques through the collection of spines as sampling material, we suggest that the European hedgehog is a relevant bioindicator species for monitoring the exposure of omnivorous terrestrial wildlife to potential toxicants in urban and rural environments.

**Abstract:**

Monitoring data from several European countries indicate that European hedgehog (*Erinaceus europaeus*) populations are declining, and research exploring the causes of the decline, including exposure to potentially harmful xenobiotics and metals, may inform conservation initiatives to protect this species in the wild. Hedgehogs are ground-dwelling mammals, feeding on a range of insects, slugs, snails, and earthworms, as well as eggs, live vertebrates, and carrion, including carcasses of apex predator species representing higher levels of the food chain. Consequently, hedgehogs come into close contact with contaminants present in their habitats and prey. This review investigated the studies available on the subject of the occurrence of metals and organic xenobiotics in hedgehogs. This study found that a vast range of different pesticides; persistent organic pollutants (POPs), including organochlorine compounds and brominated flame retardants (BFRs); as well as toxic heavy metals could be detected. Some compounds occurred in lethal concentrations, and some were associated with a potential adverse effect on hedgehog health and survival. Due to their ecology, combined with the opportunity to apply non-invasive sampling techniques using spines as sampling material, we suggest that the European hedgehog is a relevant bioindicator species for monitoring the exposure of terrestrial wildlife to potential toxicants in urban and rural environments.

## 1. Introduction

According to the United Nations Environment Programme [1] and the European Environment Agency [2], an estimated 40,000–60,000 different industrial chemicals are globally used in commerce to produce a vast range of commodities and goods, including chemical-intensive products such as computers, mobile phones, furniture, and personal care products. The European Environment Agency also estimated for 2016 that 62% of the total volume of 345 million tonnes of chemicals consumed in the European Union were hazardous to human health [2]. Several programmes have been established to monitor the occurrence of hazardous chemicals in the environment, such as the European Union Water Framework Directive [3], the Arctic Monitoring and Assessment Programme [4], or the Partnership for the Assessment of Risks from Chemicals [5]. Where biota are included, measurements often focus on the aquatic environment—in particular, fish species—whereas relatively few monitoring initiatives exist for (terrestrial) wildlife [6].

Xenobiotics are chemical substances that do not occur naturally in the organism that is studied [7]. There are several origins of xenobiotics, including industrial, household, pharmaceutical, agricultural, and transportation sources [8]. They are used in a variety of products of modern-day society and include compounds such as pharmaceuticals, personal care products, food additives, pesticides and biocides, plastic additives, and detergents [9]. Some xenobiotic compounds may have problematic properties, including toxic effects on wildlife [8]. The xenobiotics represented in this review include organochlorine industrial chemicals (e.g., polychlorinated biphenyls), brominated flame retardants (BFRs), pesticides—i.e., insecticides (including phased-out persistent ones such as DDT), rodenticides, fungicides, herbicides, nematicides, and biocides.

Metals are defined as solid substances with high electrical and thermal conductivity. They occur naturally in the lithosphere, and their compositions and concentrations vary among different localities [10]. Heavy metals are metals with relatively high atomic weights and specific densities (e.g., ≥5 g/cm^3^). In low concentrations, some metals play an essential role in maintaining various biochemical and physiological functions in living organisms (i.e., essential metals), but they become harmful when threshold concentrations are exceeded [10], similar to non-essential metals. This can lead to adverse effects on living organisms and the environment, specifically with exposure to lead, cadmium, mercury, and arsenic as the main threats [11]. Simultaneously, some metals are essential and therefore occur naturally in vertebrates such as hedgehogs [12]. These include sodium (Na), potassium (K), magnesium (Mg), calcium (Ca), iron (Fe), manganese (Mn), cobalt (Co), copper (Cu), zinc (Zn), and molybdenum (Mo), and it is currently also accepted that metal elements such as chromium (Cr) and nickel (Ni) should be included in that category, as vertebrates show certain deficiency symptoms when these metals are absent or in low concentrations [12]. Therefore, being mindful of this, distinguishing between naturally occurring low levels and elevated toxic levels remains important when interpreting results for chemically screening and detecting metals in hedgehogs.

The European hedgehog (*Erinaceus europaeus*), hereafter referred to as hedgehog, is widely distributed across Europe [13,14]. Nowadays, it primarily resides in habitats with human activity and occupation [15,16,17,18,19], including habitats with a potential exposure to xenobiotic chemicals, such as urban areas. As hedgehogs prey on a variety of insect species, earthworms, and slugs, occasionally supplementing their diet with carrion, eggs, and live vertebrate prey when available, they are potentially exposed to xenobiotic compounds from a variety of sources such as soil (topic absorption) and different types of prey species, including apex predators (carrion), by ingestion [20,21,22,23,24]. Hedgehogs have small home ranges and tend to stay in the same area throughout their lives [13,14,15]. It is therefore likely that xenobiotics in hedgehogs represent local pollution levels from the area from which the hedgehogs originated. Despite a mean suggested life expectancy of around two years (see Rasmussen et al. (2023) [25] Table 1 for an overview), hedgehogs have the potential to reach 16 years of age [25], which means they could experience long-term exposure to different xenobiotics and metals, potentially causing harmful effects in some individuals. Previous studies have found that insectivores have a greater risk of metal intoxication compared to other small mammal species like rodents [26,27]. Hedgehogs are likely exposed to metals during foraging, as they prey on a variety of insect species, earthworms, and slugs [22], all of which are known to accumulate high metal levels [28].

Hedgehogs are easy to catch and handle, and their spines and hair can be used for chemical analyses, which allows for non-destructive sampling methods [29,30]. Although these arguments make hedgehogs good candidates for monitoring programmes, few studies have focussed on their exposure to environmental xenobiotics and metals in urban and rural environments. Furthermore, substantial evidence, based on monitoring data from a range of European countries, indicates that hedgehog populations are declining [16,31,32,33,34,35,36,37,38,39,40]. The suspected causes for the decline include habitat loss; habitat fragmentation; inbreeding; intensified agricultural practices; road traffic accidents; a reduction in biodiversity and, hence, food items; lack of suitable nest sites in residential gardens; accidents caused by garden tools; netting and other anthropogenic sources in residential gardens; badger predation; and infections with pathogens and endoparasites [15,17,32,41,42,43,44,45,46,47,48,49,50,51,52,53].

Research exploring the potential causes of the decline and conservation initiatives to protect this species in the wild should consider the role of potentially harmful chemicals. Consequently, we consider it relevant to review the existing data on the occurrence of xenobiotics and metals in hedgehogs. Thus, our objective was to provide an update on the occurrence of organic xenobiotics and metals in hedgehogs, which may inspire and inform future studies on exposure to xenobiotics and metals, including their effects in hedgehogs.

## 2. Materials and Methods

To produce this literature review, the Google Scholar and Web of Science (WOS) search tools were used with these keywords: Erinaceus europaeus OR hedgehog AND a combination of 19 different search words, each entered separately, e.g., *Erinaceus europaeus* OR hedgehog AND toxicology (toxicology OR ecotoxicology OR accumulation OR xenobiotics OR bioindicator OR chemicals OR target screening OR non-target screening OR metals OR pollutants OR rodenticides OR pesticides OR herbicides OR insecticides OR molluscicides OR acaricides OR brominated flame retardants OR persistent organic pollutants OR organochlorine compounds).

**Figure 1:**
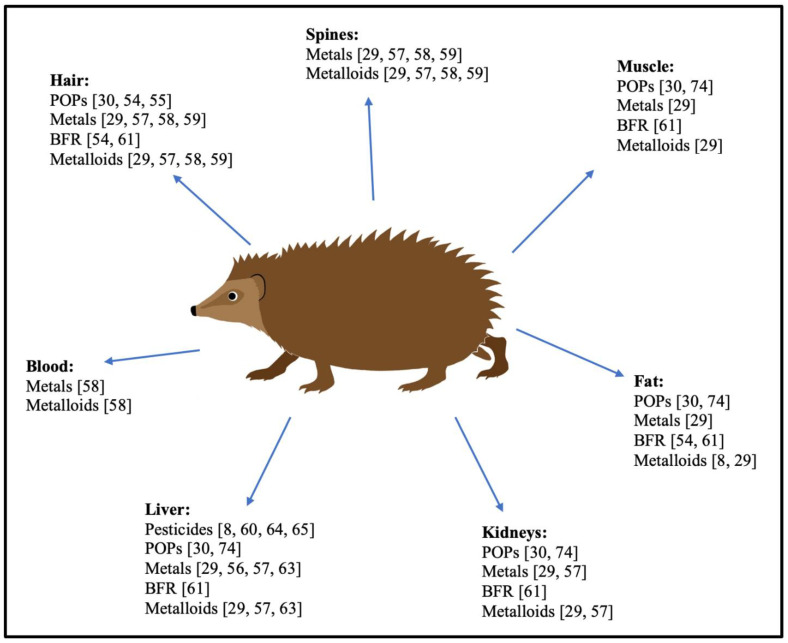
Overview of sample types used for research on xenobiotics in European hedgehogs. Abbreviations: brominated flame retardants (BFRs). Pesticides (rodenticides, insecticides, fungicides, herbicides, and nematicides) analysed: rodenticides: warfarin, coumatetralyl, difenacoum, bromadiolone, brodifacoum, flocoumafen. A total of 55 different insecticides, fungicides, herbicides, and nematicides [65]. Organochlorine compounds analysed: polychlorinated biphenyls (PCBs), dichloro-diphenyl-trichloroethanes (DDTs), hexachlorobenzene (HCB), octachlorostyrene (OCS), chlordane (CHL), hexachlorocyclohexanes (HCHs). BFRs analysed: polybrominated diphenyl ethers (PBDEs) and brominated biphenyl 153 (BB 153). Metalloids analysed: selenium and arsenic. Metals analysed: silver (Ag), aluminium (Al), cadmium (Cd), cobalt (Co), chromium (Cr), copper (Cu), iron (Fe), mercury (Hg), magnesium (Mg), manganese (Mn), molybdenum (Mo), nickel (Ni), lead (Pb), zinc (Zn).

A total of 25 results were obtained, although some were unpublished conference abstracts or reports mentioning the poisoning of hedgehogs without presenting concentration levels or specific chemicals detected [29,30,54,55,56,57,58,59,60,61,62,63,64,65,66,67,68,69,70,71,72,73,74,75,76]. These studies used a variety of sample types from hedgehogs to detect chemical compounds such as rodenticides, persistent organochlorine compounds, brominated flame retardants, metalloids, and metals. Figure 1 provides an overview of the different sample types and compounds studied. 

## 3. Results

### 3.1. Insecticides, Fungicides, Herbicides, and Nematicides

In 2021, Schanzer et al. [65] published their research screening for 55 pesticides (insecticides, fungicides, herbicides, and nematicides) in livers from six hedgehogs, dying at a wildlife rehabilitation centre in Germany. In these liver samples, the fungicides fenpropimorph and tebuconazole, the insecticides dieldrin and permethrin, as well as the metabolites fipronil sulfone (originating from the insecticide fipronil) and p,p’-DDE (originating from the persistent organic pollutant insecticide p,p’-DDT) were detected [65]. A data summary is provided in Table 1. Adding to these investigations of pesticides, Luzardo et al. (2014) [75] detected six unspecified carbamate insecticides in liver samples from six hedgehogs collected from wildlife poisoning episodes in 2010–2012. Additionally, a study reported one incidence of poisoning with the herbicide paraquat in a hedgehog in the UK and one case of poisoning of a hedgehog with the rodenticide chlorophacinone in France [72]. Carbamate insecticides and organophosphate insecticides were detected in one hedgehog, and anticoagulant rodenticides were detected in two individuals in a study on wildlife dying from suspected poisoning in Italy [73]. Gemmeke (1995) [76] experimented with the dosage of metaldehyde in live hedgehogs to determine the risk of secondary poisoning with metaldehyde. The author served 200 slugs poisoned with metaldehyde to six adult hedgehogs. Of the six hedgehogs tested, four ate all, or close to all, of the 200 slugs served, and two ate 0 and 12, respectively. None of the hedgehogs were reported to show any adverse symptoms, behavioural differences, or signs of poisoning. However, Keymer et al. (1991) [77] diagnosed metaldehyde poisoning in three dead hedgehogs collected from the UK between 1976 and 1986, and detected concentrations of up to 80 mg/kg of acetaldehyde (a by-product and metabolite of metaldehyde).

### 3.2. Rodenticides

Rodenticides are widely used and are known to accumulate within food chains, posing a threat to the survival of birds of prey and predatory mammals [78]. Given that the hedgehogs’ natural diet includes vertebrate cadavers [20,21,22,23,24], scavenging on poisoned rats and mice is not an uncommon behaviour for hedgehogs, potentially causing secondary poisoning with rodenticides. Furthermore, hedgehogs may also scavenge on carcasses of predatory species preying on rodents or ingest rodenticide pellets directly, if these are accessible to the hedgehogs in, e.g., bait boxes with holes large enough to fit a hedgehog head or by spreading the pellets directly on the ground [79,80,81]. Rodenticides are also detected in non-target invertebrates such as beetles and slugs [82,83], which constitute a considerable proportion of the natural diet of hedgehogs [22,23].

The occurrence of rodenticides in hedgehogs has been examined in a few studies described in this section. Dowding et al., 2010 [60] analysed 120 livers from hedgehogs dying in care in the UK for first-generation anticoagulant rodenticides (FGAR) and second-generation anticoagulant rodenticides (SGAR). They detected rodenticides in a total of 67% of the samples (Table 2), with a low detection frequency of flocoumafen (1/120) and the highest detection frequency of difenacoum (57/120). Detectable levels of rodenticides in liver samples from European hedgehogs ranged from 0.03 to 0.25 µg/g wet weight (Table 2). Lopéz-Perea et al. (2015) [70] screened for six anticoagulant rodenticides in liver samples from 48 hedgehogs dying in care in Spain in 2011–2013. The results showed a detection frequency ranging from 0% (warfarin) to 50% (brodifacoum), with anticoagulant rodenticides detected in 28 out of 48 individuals and a total mean concentration of 0.122 µg/g anticoagulant rodenticides detected per individual [70]. A study of livers from two hedgehogs from Spain, dying of suspected poisoning at a wildlife rehabilitation centre in 2005–2010, screened for six different rodenticides [64]. The screening detected bromadiolone (N = 2/2, mean 0.026 µg/g wet weight, range 0.013–0.049 µg/g wet weight) and brodifacoum (N = 1/2, mean 0.092 µg/g wet weight). A conference poster, presenting a study screening for anticoagulant rodenticides in six hedgehogs from Scotland collected in 2003–2013, showed a detection range of 33% and a median residue of 0.047 µg/g for unspecified anticoagulant rodenticides [69].

Based on the high levels of rodenticides detected in some of the hedgehogs, Dowding et al. (2010) [60] suggested that lethal poisoning by rodenticides was likely to occur in some hedgehogs. In the study by Sánchez-Barbudo et al. (2012) [64], it was described that, in the wildlife carcasses chosen for rodenticide screening, death was suspected to have occurred from poisoning by anticoagulant rodenticides due to discernible haemorrhages detected during necropsies, which presumably then also applied to the hedgehog carcasses included in the study, suggesting that the concentrations of rodenticides found in the two individuals were lethal. Rasmussen et al. (2019) [15] used radio tracking to monitor independent juvenile hedgehogs in the suburbs of Copenhagen (Denmark) and found one suspected case of lethal rodenticide poisoning, but the carcass was in an advanced state of decay when retrieved, preventing a chemical analysis of rodenticides. In New Zealand, where the European hedgehogs introduced are considered pests, publications have described how hedgehogs are efficiently controlled with rodenticides through the application of aerial baits and bait stations containing sodium fluoroacetate (1080) [66], which is not approved for use in European countries [84], and brodifacoum [67,68]. In one of these studies, 32 of the targeted hedgehogs were found dead and later confirmed as having been poisoned [68]. In a study conducted under laboratory conditions to investigate blood coagulation factors and the effect of warfarin, ten hedgehogs were injected with 0.4 mg/kg body weight warfarin on three consecutive days [71]. No individuals died, but the effect was a large decrease in coagulation factor activity after warfarin treatment [71]. However, even if the rodenticide doses analysed in the studies in Table 2 may not be lethal for the hedgehogs, repeated exposure, or a certain bioaccumulation, has the potential to cause different toxicological effects, which may compromise the fitness and survival of the hedgehogs. With detection frequencies of rodenticides reaching up to 99% in predatory species [81] and >90% in slugs [85], the secondary exposure to rodenticides in hedgehogs through the ingestion of poisoned prey could potentially lead to an even higher prevalence in hedgehogs than the 67% found by Dowding et al. (2010) [60].

### 3.3. Organochlorine Compounds

Many of the organochlorine compounds that have been detected in hedgehogs are regulated by the UN Stockholm Convention on persistent organic pollutants (POPs), which covers chemicals that are persistent, bioaccumulative, toxic to humans and wildlife, and can be transported over long distances [86].

The compounds that have been analysed in hedgehogs include polychlorinated biphenyls (PCBs), dichloro-diphenyl-trichloroethane (DDT and its metabolites), hexachlorobenzene (HCB), octachlorostyrene (OCS), chlordane (CHL), and hexachlorocyclohexanes (HCHs). All of these compounds, except OCS, are considered POPs according to the UN Stockholm Convention.

They were originally synthesised for industrial purposes (e.g., PCBs,) or as agrochemicals (DDT, chlordane, lindane (an HCH isomer)), while octachlorostyrene mainly forms unintentionally [87]. A common characteristic of these organochlorine compounds is their hydrophobic and lipophilic nature, leading them to bind strongly to solids such as organic matter in soil and aquatic systems and accumulate in fatty tissues of the organisms exposed to the chemicals. Lipolysis triggered by exercise can release PCBs from adipose tissue into the bloodstream [88]. As a result, the concentration, distribution, and metabolism of PCBs in plasma can differ significantly among individuals due to variations in lifestyle and behaviour.

Polychlorinated biphenyls can biomagnify [89], which has caused concern about their impact on organisms, especially on those higher up in the food chain. Exposure to organochlorine compounds can result in severe health effects, including specific cancers [90,91], birth defects, compromised immune function [92], and reproductive system dysfunction [93,94,95,96]. Additionally, it may lead to increased susceptibility to diseases [97] and damage to both the central and peripheral nervous systems [86]. Due to potential synergistic effects, it is challenging to determine the ecotoxicological influence of POPs, as a range of different POPs typically co-occur and accumulate simultaneously in biota due to their omnipresence in the environment [89].

As hedgehogs are ground-dwelling mammals, feeding on earthworms [13], they frequently come into close contact with soil, where hydrophobic organochlorines tend to accumulate, and are therefore also potentially exposed to POPs from this particular source.

Hedgehog muscles, fat, hair, livers, and kidneys have been used to study the accumulation of these organochlorine compounds [30,54,55,56] (Table 3 and Figure 1).

With sample sizes ranging from 6 to 77 individuals and sampling taking place in Belgium, the Netherlands, and Italy in 1994–2008, the different studies found a high detection frequency of organochlorine compounds in hedgehog samples in general, reaching close to 100% or 100% in many cases (Table 3), with a few exceptions in the hair samples. The occurrence in hair samples appears to be lower compared to the other sample types such as the liver, kidneys, fat, and muscle, which may be explained by the fact that these compounds accumulate in fatty tissues. The levels varied between not detected up to 31,780 ng/g dry weight and 0.1–2.8 ng/mL wet weight in blood samples. Furthermore, but not included in Table 3, the organochlorine compound p,p’-DDE (a metabolite of p,p’-DDT) was detected in three out of six hedgehog livers from Germany at concentrations ranging from 1.03 to 22.23 ng/g [65].

D’Havé et al. (2006a) [30] targeted the PCB congeners 28, 31, 74, 95, 99, 101, 105, 110, 118, 128, 138, 149, 153, 156, 163, 170, 180, 183, 187, 194, and 199 in their analyses. They found that the majority of PCB congeners in all the tissues were PCBs 153, 138/163, and 180, with a joint mean concentration of 53–63% out of the total PCB concentration in each tissue analysed.

D’Havé et al. (2007) analysed the PCB congeners 28, 31, 74, 99, 101, 105, 110, 118, 128, 138, 149, 153, 156, 170, 180, 183, 187, 194, and 199 in the hair samples from hedgehogs tested in the study. They found that PCB 118, PCB 138, and PCB 153 were dominant in the hair samples.

Vermeulen et al. (2010) [54] targeted the PCB congeners 99, 101, 118, 138, 153, 156, 170, 180, 183, and 187. Except for PCB 101, all the congeners were detected in the blood and hair samples used in the study, but PCB 118, PCB 138, PCB 153, and PCB 180 were dominant, which is in agreement with the studies by D’Havé et al. and generally presented a group of the most bioaccumulating PCB congeners. Alleva et al. (2006) [56] found that European hedgehogs had the highest levels of PCBs of the mammal species analysed in the study (mean ± SE: 6430 ± 4330 ng/g weight) and that this level was equivalent to those of insectivorous bird species, whereas the levels of fish- and small-mammal-eating bird species were considerably higher (see Table 1 in Alleva et al. (2006) [56] for an overview of the species included in the study). The authors suggested that the lower concentration of organochlorine compounds found in mammals compared to fish- and mammal-eating birds is due to the fact that mammals metabolise organochlorine compounds more readily than birds [98]. They also argued that the higher levels detected in the insectivorous species in general compared to, e.g., herbivorous species could be caused by the direct poisoning of their prey with organochlorine pollutants. Other factors to consider in the interpretation of POPs in wildlife is the sex differences found in species like the polar bear [99] and striped dolphin [100]. The POPs tend to accumulate in fat tissue, exhibiting a notable distinction between males and females in terms of a generally lower BMI index in males with more muscle mass compared to fat tissue [101]. In periods of stress, where an animal is starving, the fat is metabolised, and the accumulated POPs are then transferred from the fat to the blood stream [102,103]. However, females offload POPs via pregnancy and lactation, especially in species like the polar bear with high-nutrition milk (between 27.5 and 35.8% fat) [104,105]. However, the fat percentage in the mother’s milk of European hedgehogs is only 10% [14], which may therefore not have a similarly strong effect on the levels of POPs detected in female versus male hedgehogs. In comparison, Chu et al. (2003) [74] found that OCS concentrations in the fat tissue of the hedgehogs (mean 0.34 ng/g ww, n = 5) were similar with levels in the liver (mean 0.39 ng/g wet weight, n = 10), whereas mean HCB levels in the fat tissue (mean 20.08 ng/g lipid weight, n = 5) were markedly higher than in the liver, kidney, and muscle tissue (means ranging from 0.09 to 5.03 ng/g wet weight, n = 10–11) analysed.

### 3.4. Brominated Flame Retardants (BFRs) 

Several families of BFRs have been listed as POPs in the UN Stockholm Convention, including polybrominated diphenyl ethers (PBDEs) and hexabromobiphenyl (HBB) [86]. They were listed later than the most prominent organochlorine compounds, such as PCBs and DDT. HBB and two technical PBDE mixtures, Penta- and OctaBDE, were regulated in 2009, while the third technical PBDE product, DecaBDE, was added to the Stockholm Convention in 2017.

PBDEs and polybrominated biphenyls (PBBs) are two classes of BFRs, which have been used to improve the fire safety of synthetic polymers used in, e.g., electronic equipment, cars, building materials, and textiles [106,107]. The described toxic effects of these BRFs in vertebrates are developmental neurotoxicity, altered thyroid hormone homeostasis, liver conditions (hepatotoxicity), limb deformities in foetuses, and carcinogenic effects (tumours) [108,109]. Two studies so far have analysed the occurrence of PBDEs and the hexabrominated biphenyl BB 153 in hedgehogs [54,61] (Table 4).

Using fat, hair, kidney, liver, and muscle samples from individuals collected in Belgium and the Netherlands, with samples sizes ranging from 6 to 44, BB 153 and PBDEs were detected in all the samples, with median values of <0.10 ng/g wet weight for BB 153 and 1.2–9.5 ng/g wet weight for ∑PBDE.

D’Havé et al. (2005b) [61] reported a positive correlation between BFRs in hair and organs when considering the sum of PBDEs, concluding that hair can be used as a non-invasive alternative to organs for the monitoring of PBDE accumulation in hedgehogs. The chosen PBDE congeners 28, 47, 99, 100, 153, 154, and 183 were detected in all the sample types from the hedgehogs (hair, liver, kidney, muscle, and fat tissue) [61]. Except for the hair samples, the PBDE pattern in hedgehogs was dominated by the PBDE 47, followed by PBDEs 153 and 99. Compared with other species of wildlife, the most common PBDE congeners found in a selection of terrestrial herbivorous mammals (rabbits, moose, and reindeer) were BDEs 47, 99, and 100 [110], but, in predatory bird species, BDE 153 was the predominant congener [111,112]. These differences in the detection patterns of PDBEs between the terrestrial wildlife species may be explained by species-specific differences in PBDE metabolism and accumulation as well as food preferences, as hedgehogs and birds of prey are positioned at a higher trophic level than herbivorous species, causing diets and metabolisation to differ [112]. Furthermore, different studies were conducted in different decades, and the composition of PBDE mixtures may have changed during that period, exposing wildlife to different congeners.

### 3.5. Metals

Research has indicated that insectivores have a greater risk of metal intoxication compared to other small mammal species like rodents [26,27]. As hedgehogs prey on a variety of insect species, earthworms, and slugs [22], all of which are known to accumulate high metal levels [28], they are likely exposed to metals during foraging.

Some metals are essential and therefore occur naturally in vertebrates such as hedgehogs [12]. These include sodium (Na), potassium (K), magnesium (Mg), calcium (Ca), iron (Fe), manganese (Mn), cobalt (Co), copper (Cu), zinc (Zn), and molybdenum (Mo), and it is currently also accepted that metal elements such as chromium (Cr) and nickel (Ni) should be included in that category, as vertebrates show certain deficiency symptoms when these metals are absent or in low concentrations [12]. Therefore, being mindful of this, distinguishing between naturally occurring low levels and elevated toxic levels remains important when interpreting results from chemical screening and detection of metals in hedgehogs.

In our literature search, we found six studies that investigated the presence of metals in hedgehogs, using sample material ranging from hair; spines; and tissues such as kidney, liver, fat, and muscle to blood [29,56,57,58,59,63]. The metals tested were silver (Ag), aluminium (Al), cadmium (Cd), cobalt (Co), chromium (Cr), copper (Cu), iron (Fe), mercury (Hg), magnesium (Mg), manganese (Mn), molybdenum (Mo), nickel (Ni), lead (Pb), and zinc (Zn) (see Supplementary Materials for an overview).

The detection of metals was based on samples of blood, spines, livers, muscles, kidneys, hair, and fat, with sample sizes between 7 and 83. The levels detected (blood samples excluded) were Ag (ND–0.62 µg/g, mean ND–0.12 µg/g), Al (ND–230 µg/g, mean 8–76 µg/g), Cd (ND–337 µg/g, mean 0.04–45.17 µg/g), Co (ND–1366 µg/g, mean 0.01–0.99 µg/g), Cr (0–30.9 µg/g, mean 0–5.4 µg/g), Cu (0.2–200 µg/g, mean 1.8–64 µg/g), Fe (ND–2849.76 µg/g, mean 22.94–2339 µg/g), Hg (0.19 µg/g, mean 0.06 µg/g), Mg (46.33–1086.24 µg/g, mean 144.88–731.04 µg/g), Mn (ND–31.11 µg/g, mean 1.85–6.33 µg/g), Mo (ND–5.12 µg/g, mean ND–2.55 µg/g), Ni (ND–35 µg/g, mean 0.07–0.73 µg/g), Pb (ND–31.5 µg/g, mean 0.54–10.9 µg/g), and Zn (0.1–7.47 µg/g, mean 0.06–228.97µg/g).

Including information on the age of the individuals in their study, Rautio et al. (2010) [57] found significant increases in the levels of several metals (Cd, Mo, Cu, Fe, Mn) with increasing age, although this depended on the tissue types analysed, suggesting an age-related bioaccumulation of metals in hedgehogs. This was supported by Jota Baptista et al. (2023) [63], showing that concentrations of Cd and Co were significantly lower in juvenile compared to adult individuals. At present, it appears that no values describing physiologically normal concentrations of essential metals exist for European hedgehogs, which complicates an interpretation and discussion of the concentrations of essential metals in hedgehogs. However, Jota Baptista et al. (2023) [63] detected biliary hyperplasia in 16 of the 45 hedgehogs examined, concluded that concentrations of metals were higher in individuals with this condition, and suggested that heavy metals and metalloids may be the primary contributing factor causing biliary hyperplasia in hedgehogs.

### 3.6. Selenium and Arsenic Metalloids in Hedgehogs

Selenium is an essential trace element and has several important functions in the metabolism of animals, e.g., as an antioxidant constituting a component of glutathione peroxidase (GSHPx), assisting in intracellular defence mechanisms against oxidative damage [113]. However, selenium poisoning, or selenosis, has been described in production animals through conditions called “blind staggers” and “alkali disease” [114], causing impaired vision, ataxia, and deformities in nails, hooves, and hair [114,115,116]. However, research also suggests that selenoproteins and other selenium metabolites are important in regulating immune function and reducing cancer risk [117]. Selenium deficiency is known to cause a range of health conditions in vertebrates [118,119], which is why selenium is used extensively in fertilizers, especially as an enrichment of livestock feed crops [120]. Natural sources of selenium include marls, gypsum, volcanic eruptions, sea spray, and the weathering of rocks and soils containing selenium. Anthropogenic sources, constituting the majority of the influx of selenium to the environment, include mining, agriculture, coal combustion, insecticide production, oil refining, photocells, and glass production [119]. Industrial and agricultural activities are the dominant anthropogenic sources of selenium pollution in, e.g., soil and drinking water [119].

Arsenic is a widespread element occurring worldwide [121], which originates from natural geogenic sources, as it is a major constituent of more than 245 minerals [122], and from anthropogenic sources. Anthropogenic activities contribute three times as much as natural sources to the accumulation of arsenic in the environment [122]. Out of these, industrial effluents constitute the largest contribution. Most of the arsenic is used for the preservation of wood, but the manufacturing of paints, dyes, ceramics and glass, electronics, pigments, and antifouling agents also include arsenic. Agricultural inputs from chemicals such as insecticides, herbicides, desiccants, and fertilizers are a major source of arsenic in soils. Insecticidal products containing arsenic have previously been used extensively for the treatment of ectoparasites in livestock [123].

Arsenic appears in several chemical forms, all with different degrees of toxicity. Inorganic forms of arsenic (arsenite and arsenate) are more toxic, while methylated forms (methylarsonate (MMA) and dimethylarsinate (DMA)) are moderately toxic [121]. Other arsenic species, like trimethyl-arsine oxide (TMAO) and tetramethyl-arsonium (TETRA) are also considered moderately toxic. By contrast, the forms arsenobetaine (AsB), arsenocholine (AsC), and other arsenosugars (AsS) appear to have low or very low toxicity [124]. The toxicity caused by arsenic exposure is linked to an imbalance between pro-oxidant and antioxidant homeostasis, which results in oxidative stress [125]. The general mechanism behind the toxic effects of arsenic is the oxidative deterioration of polyunsaturated fatty acids, a process known as lipid peroxidation [126]. Research on the health aspects of arsenic exposure has revealed how chronic exposure may cause cancer in the skin, lungs, bladder, and liver [127].

Given the potential toxicity of arsenic and selenium exposure, and the presence of these metalloids in the soil and water, it is relevant to explore the occurrence and bioaccumulation of these compounds in hedgehogs. Five different studies so far have addressed the prevalence of the specific metalloids selenium [57] and arsenic [29,57,58,59,63] in hedgehogs. Table 5 provides an overview of the findings.

The sampling took place in Finland, Belgium, and the Netherlands in the years 2002–2006 and in Portugal in 2019–2021, with sample sizes ranging from 7 to 83 hedgehogs, representing the sample types of hair, kidney, liver, spine, muscle, blood, and fat. For selenium, the detection levels ranged between not detected to 11.36 µg/g dry weight, with means ranging from 0.18 to 4.63 µg/g. Arsenic had detection levels from ND to 23.6 µg/g in the hair, kidneys, livers, spines, muscles, and fat, with means ranging from 0.08 to 1.24 µg/g. In the blood samples, levels of arsenic ranged from 0.1 to 155.5 µg/mL. The data presented in the studies unfortunately did not allow for a representation of detection frequencies for the two metalloids.

Rautio et al. (2010) [57] found that selenium concentrations increased significantly with increasing age in all the tissue types studied, suggesting a gradual accumulation of this compound in hedgehogs with age. Jota Baptista et al. (2023) [63] described how levels of arsenic were significantly lower in independent juveniles compared to adults and dependent juveniles.

## 4. Discussion

### 4.1. More Research on Exposure to Xenobiotics

Hedgehogs are increasingly inhabiting areas of human occupation [15,16,17,18,19], such as residential gardens and urban parks, where they navigate through dense shrubberies, flower beds, vegetable gardens, and open green spaces. Facilitated by their short stature, rarely reaching > 15 cm in height, they may be exposed to many sources of herbicides and insecticides, as they come into close contact with plants during foraging, in addition to consuming prey items living and feeding on these plants, which are then targeted by insecticides [20,22,23,128]. Dietary studies have also revealed remnants of plants and fruit in the stomachs of hedgehogs, although it is unknown whether they intentionally fed on the plants or whether they were ingested during an attempt to catch prey items positioned on the plant material [13,20]. The use of insecticides in residential gardens serving to eliminate species of, e.g., ants and aphids, is common. Hedgehogs may be exposed to insecticides through foraging on poisoned prey items but also by moving through treated shrubs and areas (in the case of aerosol or liquid insecticidal products) or by ingesting the poison through oral intake, if the poison is placed in the open.

Hedgehog populations in rural areas appear to face the highest decline [37,39], including agricultural landscapes, with a possible pathway of exposure during foraging to herbicides and insecticides used in cultivated fields [129].

Hedgehogs admitted into care at a wildlife rehabilitation centre may furthermore become exposed to insecticides through flea treatments in cases where their ectoparasite burdens have become extensive enough to cause a reduction in fitness, such as anaemia, requiring treatment. Evidence from wildlife rehabilitation centres has led to a general consensus among hedgehog rehabilitators and veterinarians treating hedgehogs that permethrin is likely lethal to hedgehogs, as it is to cats [130], with its documented critical effect concentration (PNEC) of 120 mg permethrin/kg food for small mammals [131] and an oral lethal dose of 50 (LD_50_) for rats of 480 mg permethrin/kg bodyweight [131], with an estimated LD_50_ of 480 mg permethrin/kg bodyweight for mammals in general, in case of primary poisoning [132]. But are hedgehogs otherwise exposed to insecticides intended for the treatment of ectoparasites in pets? As these substances are excreted from dogs and cats through urine and faeces [133,134,135], there is a risk that hedgehogs may come into close contact with the compounds, as they sometimes cover themselves in faeces from dogs and cats, likely as an attempt to disguise their own smell for predators. Furthermore, sharing sources of fresh water with pets could also lead to an exposure of insecticides used for treatment against endoparasites, if a dog swims or rolls in a small puddle or a lake or stream from which the hedgehog drinks, as a range of the products against ectoparasites are nowadays “spot on” products, which are applied directly onto the skin and fur of the pets [136]. Schanzer et al. (2021) [65] detected permethrin in one individual and a metabolite of fipronil in all of the six hedgehogs analysed, which may indicate that exposure to insecticidal treatments for ectoparasites in cats and dogs is widespread in hedgehogs.

### 4.2. Food and Waterborne Contaminants

As hedgehogs are frequently offered supplementary feeding with commercial cat food in residential gardens [137], it would also be relevant to analyse cat food for different potentially toxic compounds, as recent research discovered that perfluoroalkylated substances (PFASs) were found, especially in organic chicken eggs, likely due to the addition of fish meal in commercial chicken feed [138,139]. Fish meal is also a common ingredient in commercial cat and dog food [140], which may cause PFASs to accumulate in hedgehogs feeding on these products, in addition to the exposure of PFAS from other sources, such as contaminated sites and wastewater [141]. Even though the toxic effects of PFASs are currently unknown for hedgehogs, previous research indicates negative health impacts of the bioaccumulation of PFASs in wildlife, e.g., a significant association between infectious diseases and elevated concentrations of perfluorooctanesulfonate (PFOS) and perfluorooctanoic acid (PFOA) in the livers of southern sea otters (*Enhydra lutris nereis*) [142].

Municipal wastewater is well known to contain a range of xenobiotics, including pesticides, PFAS, and flame retardants but also pharmaceutical products [143], excreted through urine and faeces from their human users. Sewage sludge is also a common fertiliser that contains a range of compounds such as heavy metals [144], pharmaceuticals [145], and pesticides [146]. The same applies to manure from livestock treated with different types of medical drugs. When sludge or manure is spread as fertilisers on the fields in which hedgehogs forage, a possible exposure pathway for hedgehogs is created. Furthermore, there may also be residues of pesticides, PFAS, pharmaceuticals, and other pollutants in the plain drinking water [147] provided for hedgehogs in people’s gardens in, e.g., ponds and water bowls.

### 4.3. Non-Target Screening

Traditionally, analytical chemistry applies trace-level chemical analytical methods for a specific type of sample and group of substances. This form of targeted analysis is used for the identification and quantification of specific compounds, especially at low levels. However, target analyses only identify compounds that have been defined in advance, potentially overlooking other compounds with toxic potential. New analytical techniques, such as non-target screening based on high resolution mass spectrometry (HRMS), offer a possibility to scan for unknown compounds in a sample (e.g., Hollender et al. (2017) [148]). HRMS can be coupled with different types of chromatographic separation methods, i.e., liquid chromatography (LC) and gas chromatography (GC), for polar and non-polar compounds, respectively. Thus, a combination of both will be required to cover a broad spectrum of organic chemicals [149]. Following the recording of high-resolution mass spectra, bioinformatic analysis is applied to identify the compounds via comparisons with mass spectra libraries. Non-target screening would be a useful complementary approach for research on xenobiotics in hedgehogs, as it would allow for a more comprehensive screening of substances in hedgehog samples, providing insights into potentially overlooked compounds. If analytical standards are available for the identified compound, a target method for quantification can be developed as a second step. Due to the potentially high number of chemicals hedgehogs can be exposed to, we would like to advocate for the use of non-target screening in future studies on xenobiotics in hedgehogs.

### 4.4. The Health- and Age-Related Effects of the Occurrence of Contaminants in Hedgehogs

So far, research has primarily focused on quantifying the extent of contaminants in hedgehogs, detecting levels and frequencies of toxic, and potentially lethal, compounds but has, until recently (2023), not related these exposure levels to health effects in hedgehogs. However, Jota Baptista et al. (2023) [63] detected biliary hyperplasia in 16 of the 45 dead hedgehogs examined in their study and concluded that concentrations of metals were higher in individuals with biliary hyperplasia. We encourage future studies to investigate the potential toxicological effects of the widespread occurrence of rodenticides, organochlorine compounds, and BFRs in this declining species and expand this to currently understudied compounds, such as insecticides and PFAS. Since hedgehogs are exposed to multiple compounds at the same time, their combined effects will be relevant to address as well. Rautio et al. (2010) [57] found evidence of an age-related increase in concentrations of different metals (Cd, Se, Mo, Cu, Fe, and Mn) in hedgehogs, which is also a relevant subject in need of further study, including the health effects of bioaccumulation of multiple metals, especially given the fact that European hedgehogs have the potential to reach 16 years of age [25].

### 4.5. Selecting the Relevant Sample Types

The published studies investigated in this review used different approaches and sample types for studying the occurrence of xenobiotics and metals in hedgehogs, including spines, hair, muscles, fat, livers, kidneys, and blood (Figure 1). Depending on the compound in focus, its physical–chemical characteristics, and physiological processes, some sample types seemed more representative than others. As an example, Vermeulen et al. (2009) [58] found that the levels of As, Cd, and Pd were correlated in the hair, spines, and blood, but, by contrast, this did not apply to Al, Cr, Cu, Fe, Mn, Ni, or Zn. D’Havé et al. (2006B) [29] discovered the highest concentrations of metals in internal tissues compared to the hair and spines, with Ag, Fe, Pb, and Zn concentrations being dominant in the livers, and Cd and Co measured in the highest levels in liver and kidney tissue. Furthermore, the authors concluded that external tissues, such as the hair and spines, may accumulate substantial concentrations of certain metals (Al, Cr, Cu, and Ni) and As. They recorded the highest concentrations for Al in spines, while As was predominant in the hair and spines. Rautio et al. (2010) [57] found that As, Cd, and Se concentrations were the highest in the kidneys, compared to Fe, Mg, Mn, Mo, Pb, and Zn, which were the highest in the livers, and Cu and Ni levels being the highest in the hair. In this study, there was a general tendency for the concentrations of the chemical compounds investigated to be lower in spine samples compared to samples from internal organs [57]. Lipophilic compounds typically accumulate in lipid-rich tissues, and the concentrations of liposoluble toxicants may vary due to morphological and behavioural differences between the sexes.

We encourage harmonised approaches for monitoring purposes, including an alignment of protocols regarding tissue types selected for analyses and sampling techniques, as well as quality control measures for the harmonisation of analytical methods. These combined efforts would improve the comparability of the results. However, while the standardisation of tissue types is important, there is also a need for analyses of different organs and tissues to improve the toxicokinetic understanding of xenobiotics and metals in hedgehogs.

### 4.6. Non-Destructive Measures and Hibernating Mammals as Bioindicators

Several of the research papers reviewed suggest that hedgehogs may serve as potential bioindicators for studies on the presence and accumulation of different environmental pollutants, as they share habitats with a wide range of vertebrates, and their spines appear to be a valuable and non-invasive sample type for the analysis of selected chemicals. However, for a correct interpretation of the detection of chemical compounds, a better understanding of the metabolism of contaminants in hedgehogs would be useful. It should also be considered that the direct causes of exposure to chemicals in humans and hedgehogs are not necessarily identical even though they share habitats, as humans generally do not tend to eat insects in Europe. Instead, humans may eat the same plants as the insects, which are then consumed by the hedgehogs. However, signals of potentially harmful compounds in hedgehog samples may indicate exposure sources in specific areas that would benefit from closer investigation to prevent or reduce exposure to other species.

Using spines from hedgehogs may serve as an important non-invasive alternative to traditional organ analyses of sacrificed animals [150]. The spines can be collected through a non-invasive method, as they do not contain any nerves [13], and can be sampled very rapidly with a minimum duration of handling, potentially only causing a low degree of acute stress to the hedgehog being sampled [151]. However, it should be considered that the concentrations and chemical compounds found in spines are not necessarily directly comparable to those found in organs [57,58]. Additionally, the use of dead hedgehogs for research collected by volunteers in the wild is also widely applied, and citizen science projects like The Danish Hedgehog Project have provided large numbers of samples from a wide range of habitat types for a variety of different research purposes [25,47,48,50,51]. The public adoration of hedgehogs makes large-scale citizen science projects possible, where the use of dead hedgehogs collected in the wild could also serve as a non-invasive sampling technique for future studies of xenobiotic exposure and ecotoxicology.

In contrast to actively wintering small mammals that are forced to increase their food intake during colder temperatures, potentially leading to a higher exposure of pollutants during the winter, hedgehogs hibernate for up to six months a year in most of their geographical distribution [13,14]. This may influence the accumulation of xenobiotics in their tissues. The potential lack of metabolisation of different chemical compounds during the state of torpor in hedgehogs could perhaps affect the levels detected in hedgehogs compared to non-hibernating species. Additionally, they are also likely to be affected by “delayed toxicity” through the metabolisation of adipose tissue with accumulated pollutants during hibernation.

Therefore, we advocate for research investigating these potential influences on the levels of xenobiotics and metals detected in hedgehogs compared to other small mammal species, enabling a more robust comparison between future studies with hedgehogs utilised as bioindicator species.

## 5. Conclusions

This review aimed to provide a comprehensive overview of the available studies screening for xenobiotics and metals in hedgehogs. Our findings revealed that a vast range of different pesticides, POPs, including organochlorine compounds and BFRs, metals, and metalloids, could be detected in samples from hedgehogs collected from different locations throughout Europe. In some cases, the compounds reached lethal concentrations, causing fatal poisoning in hedgehogs, and, in other cases, adverse health impacts, such as biliary hyperplasia, were described in the poisoned hedgehogs. Since some studies included animals that had died from poisoning, it is important to note that these might present a bias towards high concentrations, rather than representing general exposure levels. Moreover, given the lack of information on lethal doses for European hedgehogs, the interpretation of the concentrations of xenobiotics and metals present in the hedgehogs with regard to toxic effects is challenging and restricts us to drawing conclusions about the presence of these compounds in the hedgehogs.

Because we share habitats, toxicological screenings of hedgehogs could also indicate the potential exposure of xenobiotics to other terrestrial vertebrates. Hedgehogs are ground-dwelling mammals, feeding on a range of insects, slugs, snails, and earthworms and thereby come into close contact with contaminants present in the soil. They also feed on carrion, potentially accumulating compounds found in higher levels of the food chain from apex predator species. Combined with the opportunity to apply non-invasive sampling techniques through the collection of spines as sampling material, as well as the large potential for citizen science projects collecting dead hedgehogs in the wild, the European hedgehog should be regarded as a relevant bioindicator species. Furthermore, hedgehogs are declining in Europe, and insights gained through research on the role of xenobiotics and heavy metals in this decline will help inform future conservation actions directed at this species.

Due to this important potential, we advocate for more research into the exposure to and potential bioaccumulation of xenobiotics and metals in hedgehogs with a standardisation and harmonisation of sampling techniques, sample types, and methods of analysis in future studies, which would be imperative for facilitating robust comparisons. Additionally, incorporating non-target screening techniques will enable the detection of hitherto overlooked relevant and potentially toxic substances.

## Figures and Tables

**Table 1:** Results from the screening for insecticides, fungicides, herbicides, and nematicides in liver samples from German hedgehogs by Schanzer et al. (2021) [65]. The sampling material was six livers from German hedgehogs dying in care, and they were analysed by gas chromatography coupled to tandem mass spectrometry (GC-MS/MS).

Compound	Pesticide Type	Levels Detected µg/g	Frequency of Positives %	Frequency of Positives N/n
p,p’-DDE	Metabolite of the insecticide p,p’-DDT	0.001–0.22	50	3/6
Fenpropimorph	Fungicide	0.0005–0.002	66.67	4/6
Fipronil sulfone	Insecticide	0–0.05	100	6/6
Tebuconazole	Fungicide	0–0.008	33.33	2/6
Dieldrin	Insecticide	0.003–0.0031	16.67	1/6
Permethrin	Insecticide	0.007–0.0073	16.67	1/6

**Table 2:** Results from the screening for rodenticides in liver samples from hedgehogs. Abbreviations used: first-generation anticoagulant rodenticides (FGAR), second-generation anticoagulant rodenticides (SGAR), United Kingdom (UK), Spain (S), not applicable (na). All the hedgehogs tested had died in care. All the analyses were based on liquid chromatography (LC), with either fluorescence or mass spectrometric detection.

Reference	Compound	Type	Sample Size	Sampling Year	Country	Median Levels Detected (μg/g Wet Weight)	Mean Levels Detected (μg/g Wet Weight)	Frequency %	Frequency N/n
Dowding et al., 2010 [60]	Warfarin	FGAR	120	2004–2006	UK	0.03		8	10/120
Dowding et al., 2010 [60]	Coumatetralyl	FGAR	120	2004–2006	UK	0.05		14	17/120
Dowding et al., 2010 [60]	Difenacoum	SGAR	120	2004–2006	UK	0.05		13	16/120
Dowding et al., 2010 [60]	Difenacoum	SGAR	120	2004–2006	UK	0.06		48	57/120
Dowding et al., 2010 [60]	Bromadiolone	SGAR	120	2004–2006	UK	0.25		11	13/120
Dowding et al., 2010 [60]	Bromadiolone	SGAR	120	2004–2006	UK	0.05		19	23/120
Dowding et al., 2010 [60]	Brodifacoum	SGAR	120	2004–2006	UK	0.06		5	6/120
Dowding et al., 2010 [60]	Brodifacoum	SGAR	120	2004–2006	UK	0.04		3	4/120
Dowding et al., 2010 [60]	Flocoumafen	SGAR	120	2004–2006	UK	na		1	1/120
Dowding et al., 2010 [60]	Flocoumafen	SGAR	120	2004–2006	UK	na		0	0/120
Dowding et al., 2010 [60]	Total SGAR	SGAR	120	2004–2006	UK	na		58	69/120
Dowding et al., 2010 [60]	Total SGAR	SGAR	120	2004–2006	UK	na		23	27/120
Dowding et al., 2010 [60]	Total FGAR and SGAR	FGAR + SGAR	120	2004–2006	UK	na		67	80/120
Sánchez-Barbudo et al., 2012 [64]	Bromadiolone	SGAR	2	2005–2010	S		0.026	100	2/2
Sánchez-Barbudo et al., 2012 [64]	Brodifacoum	SGAR	2	2005–2010	S		0.092	50	1/2
Lopéz-Perea et al., 2015 [70]	Warfarin	FGAR	48	2011–2013	S		ND	0	0/48
Lopéz-Perea et al., 2015 [70]	Coumatetralyl	FGAR	48	2011–2014	S		0.08	27	13/48
Lopéz-Perea et al., 2015 [70]	Difenacoum	SGAR	48	2011–2015	S		0.008	25	12/48
Lopéz-Perea et al., 2015 [70]	Bromadiolone	SGAR	48	2011–2016	S		0.0074	12.5	6/48
Lopéz-Perea et al., 2015 [70]	Brodifacoum	SGAR	48	2011–2017	S		0.043	50	24/48
Lopéz-Perea et al., 2015 [70]	Flocoumafen	SGAR	48	2011–2018	S		0.023	4	2/48

**Table 3:** Results from studies investigating organochlorine compounds in European hedgehogs. Abbreviations used: Belgium (BE), the Netherlands (NL), Italy (I), not detected (ND), not applicable (na), polychlorinated biphenyls (PCBs), dichloro-diphenyl-trichloroethane and metabolites (DDTs), hexachlorobenzene (HCB), octachlorostyrene (OCS), chlordane (CHL), hexachlorocyclohexanes (HCHs). Unknown is written when the number of samples is unknown and when it is unknown whether only positive samples were represented in the dataset. All the samples were analysed with gas chromatograph (GC)–mass spectrometry (MS). * denotes a range of medians for samples representing seven study sites.

Reference	Com-pound	Sample Material	Sample Size	Sampling Year	Country	Levels Detected (ng/g Dry Weight)	Levels Detected (ng/mL Wet Weight)	Median Levels Detected (ng/g Dry Weight)	Hedgehogs Analysed	Frequency of Positives %	Frequency of Positives N/n
**CHL**											
D’Havé et al., 2006a [30]	CHL	Fat	6	2002–2003	BE + NL	1.9–14.1	na	4.7	Dead, roadkill, and dead in care	100	6/6
D’Havé et al., 2006a [30]	CHL	Hair	45	2002–2003	BE + NL	ND–1.2	na	ND	Dead, roadkill, and dead in care	33	15/45
D’Havé et al., 2006a [30]	CHL	Kidney	44	2002–2003	BE + NL	0.02–26.4	na	0.5	Dead, roadkill, and dead in care	100	44/44
D’Havé et al., 2006a [30]	CHL	Liver	43	2002–2003	BE + NL	0.2–75.7	na	5.1	Dead, roadkill, and dead in care	100	43/43
D’Havé et al., 2006a [30]	CHL	Muscle	44	2002–2003	BE + NL	0.01–20.7	na	0.5	Dead, roadkill, and dead in care	100	44/44
**DDT**											
Vermeulen et al., 2010 [54]	DDTs	Blood	13	2005–2007	BE	na	0.1–0.4	na	Wild, live hedgehogs	Unknown	Unknown
D’Havé et al., 2006a [30]	DDTs	Fat	6	2002–2003	BE + NL	5.51–194	na	18.1	Dead, roadkill, and dead in care	100	6/6
Alleva et al., 2006 [56]	DDTs	Fat	Unknown	1994–1995	I	0–27,680	na	1490	Dead, roadkill	na	na
Vermeulen et al., 2010 [54]	DDTs	Hair	18	2005–2006	BE	0.2–5.7	na	na	Wild, live hedgehogs	Unknown	Unknown
D’Havé et al., 2006a [30]	DDTs	Hair	45	2002–2003	BE + NL	ND–725	na	2.5	Dead, roadkill, and dead in care	91	41/45
D’Havé et al., 2007 [55]	DDTs	Hair	77	2002	BE	ND–84	na	ND–0.9	Wild, live hedgehogs	60	46/77
D’Havé et al., 2006a [30]	DDTs	Kidney	44	2002–2003	BE + NL	ND–1313	na	1.8	Dead, roadkill, and dead in care	98	43/44
D’Havé et al., 2006a [30]	DDTs	Liver	43	2002–2003	BE + NL	ND–750	na	1.4	Dead, roadkill, and dead in care	98	42/43
D’Havé et al., 2006a [30]	DDTs	Muscle	44	2002–2003	BE + NL	0.02–1444	na	2.3	Dead, roadkill, and dead in care	100	44/44
**HCB**											
D’Havé et al., 2006a [30]	HCB	Fat	6	2002–2003	BE + NL	1.6–67.2	na	4.42	Dead, roadkill, and dead in care	100	6/6
D’Havé et al., 2006a [30]	HCB	Hair	45	2002–2003	BE + NL	0.02–334.7	na	0.16	Dead, roadkill, and dead in care	78	35/45
D’Havé et al., 2007 [55]	HCB	Hair	77	2002	BE	ND–679	na	ND–40.7	Wild, live hedgehogs	55	42/77
D’Havé et al., 2006a [30]	HCB	Kidney	44	2002–2003	BE + NL	0.02–160.1	na	0.26	Dead, roadkill, and dead in care	100	44/44
D’Havé et al., 2006a [30]	HCB	Liver	43	2002–2003	BE + NL	0.02–247.6	na	0.28	Dead, roadkill, and dead in care	100	43/43
D’Havé et al., 2006a [30]	HCB	Muscle	44	2002–2003	BE + NL	0.04–135.3	na	0.31	Dead, roadkill, and dead in care	100	44/44
Chu et al., 2003 [74]	HCB	Fat	5	2001–2002	BE	1.61–82.54	na	20.08	Unknown	na	na
Chu et al., 2003 [74]	HCB	Liver	10	2001–2002	BE	0.11–4.49	na	1.27	Unknown	na	na
Chu et al., 2003 [74]	HCB	Muscle	11	2001–2002	BE	0.09–5.03	na	0.97	Unknown	na	na
Chu et al., 2003 [74]	HCB	Kidney	11	2001–2002	BE	0.09–4.65	na	0.96	Unknown	na	na
**HCHs**											
D’Havé et al., 2006a [30]	HCHs	Fat	6	2002–2003	BE + NL	1.1–2.4	na	1.4	Dead, roadkill, and dead in care	100	6/6
D’Havé et al., 2006a [30]	HCHs	Hair	45	2002–2003	BE + NL	ND–105.5	na	0.7	Dead, roadkill, and dead in care	93	42/45
D’Havé et al., 2007 [55]	HCHs	Hair	77	2002	BE	ND–134.8	na	ND–12.8	Wild, live hedgehogs	70	54/77
D’Havé et al., 2006a [30]	HCHs	Kidney	44	2002–2003	BE + NL	0.03–2.9	na	0.2	Dead, roadkill, and dead in care	100	44/44
D’Havé et al., 2006a [30]	HCHs	Liver	43	2002–2003	BE + NL	ND–8.8	na	0.1	Dead, roadkill, and dead in care	98	42/43
D’Havé et al., 2006a [30]	HCHs	Muscle	44	2002–2003	BE + NL	ND–11.5	na	0.2	Dead, roadkill, and dead in care	98	43/44
**OCS**											
D’Havé et al., 2006a [30]	OCS	Fat	6	2002–2003	BE + NL	0.08–0.5	na	0.4	Dead, roadkill, and dead in care	100	6/6
D’Havé et al., 2006a [30]	OCS	Hair	45	2002–2003	BE + NL	ND–0.06	na	ND	Dead, roadkill, and dead in care	9	4/45
D’Havé et al., 2006a [30]	OCS	Kidney	44	2002–2003	BE + NL	0.01–1.1	na	0.1	Dead, roadkill, and dead in care	100	44/44
D’Havé et al., 2006a [30]	OCS	Liver	43	2002–2003	BE + NL	0.03–3.1	na	0.2	Dead, roadkill, and dead in care	100	43/43
D’Havé et al., 2006a [30]	OCS	Muscle	44	2002–2003	BE + NL	0.01–0.9	na	0.1	Dead, roadkill, and dead in care	100	44/44
Chu et al., 2003 [74]	OCS	Fat	5	2001–2002	BE	0.08–0.49	na	0.34	Unknown	na	na
Chu et al., 2003 [74]	OCS	Liver	10	2001–2002	BE	0.14–1.10	na	0.39	Unknown	na	na
Chu et al., 2003 [74]	OCS	Muscle	11	2001–2002	BE	0.01–0.29	na	0.08	Unknown	na	na
Chu et al., 2003 [74]	OCS	Kidney	11	2001–2002	BE	0.01–0.32	na	0.12	Unknown	na	na
**PCB**											
Vermeulen et al., 2010 [54]	PCB	Blood	13	2005–2009	BE	na	0.2–2.8	na	Wild, live hedgehogs	Unknown	Unknown
D’Havé et al., 2006a [30]	PCB	Fat	6	2002–2003	BE + NL	89–739	na	273	Dead, roadkill, and dead in care	100	6/6
Alleva et al., 2006 [56]	PCB	Fat	Unknown	1994–1995	I	0–31,780	na	1800	Dead, roadkill	na	na
Vermeulen et al., 2010 [54]	PCB	Hair	18	2005–2008	BE	0.6–13.5	na	na	Wild, live hedgehogs	Unknown	Unknown
D’Havé et al., 2006a [30]	PCB	Hair	45	2002–2003	BE + NL	ND–789	na	10	Dead, roadkill, and dead in care	96	43/45
D’Havé et al., 2007 [55]	PCB	Hair	77	2002	BE	ND–65	na	1–5 *	Wild, live hedgehogs	69	53/77
D’Havé et al., 2006a [30]	PCB	Kidney	44	2002–2003	BE + NL	3–5150	na	49	Dead, roadkill, and dead in care	100	44/44
D’Havé et al., 2006a [30]	PCB	Liver	43	2002–2003	BE + NL	2–5910	na	75	Dead, roadkill, and dead in care	100	45/45
D’Havé et al., 2006a [30]	PCB	Muscle	44	2002–2003	BE + NL	5–2940	na	50	Dead, roadkill, and dead in care	100	44/44

**Table 4:** An overview of the results from studies on brominated flame retardants (BFRs) in European hedgehogs. Abbreviations used: polybrominated diphenyl ethers (PBDEs), brominated biphenyl 153 (BB 153), the Netherlands (NE), Belgium (BE), not applicable (na). All the samples were analysed with gas chromatography–mass spectrometry (GC-MS).

Reference	Compound	Sample Type	Sample Size	Sampling Year	Country	Levels Detected (ng/g Wet Weight)	Median Levels Detected (ng/g Wet Weight)	Hedgehogs Analysed
**BB 153**								
D’Havé et al., 2005b [61]	**BB 153**	Fat	6	2002–2003	BE + NL	<0.10–0.2	<0.10	Roadkill and dead in care
D’Havé et al., 2005b [61]	**BB 153**	Hair	32	2002–2003	BE + NL	<0.05–0.6	0.09	Roadkill and dead in care
D’Havé et al., 2005b [61]	**BB 153**	Kidney	44	2002–2003	BE + NL	<0.10–1.1	<0.10	Roadkill and dead in care
D’Havé et al., 2005b [61]	**BB 153**	Liver	43	2002–2003	BE + NL	<0.10–2.5	<0.10	Roadkill and dead in care
D’Havé et al., 2005b [61]	**BB 153**	Muscle	44	2002–2003	BE + NL	<0.10–1.1	<0.10	Roadkill and dead in care
**PBDE**								
D’Havé et al., 2005b [61]	**PBDE**	Fat	6	2002–2003	BE + NL	3.1–19.4	9.1	Roadkill and dead in care
Vermeulen et al., 2010 [54]	**PBDE**	Hair	18	2005–2010	BE	0.01–3.3	na	Wild, live hedgehogs
D’Havé et al., 2005b [61]	**PBDE**	Hair	32	2002–2003	BE + NL	0.8–11	1.5	Roadkill and dead in care
D’Havé et al., 2005b [61]	**PBDE**	Kidney	44	2002–2003	BE + NL	0.4–238.9	1.2	Roadkill and dead in care
D’Havé et al., 2005b [61]	**PBDE**	Liver	43	2002–2003	BE + NL	1–1177.5	9.5	Roadkill and dead in care
D’Havé et al., 2005b [61]	**PBDE**	Muscle	44	2002–2003	BE + NL	0.3–316.3	1.5	Roadkill and dead in care

**Table 5:** An overview of the prevalence and detection levels of the metalloids selenium and arsenic in European hedgehogs. Abbreviations used: Belgium (BE), the Netherlands (NL), Finland (FI), Portugal (PT), not detected (ND), not applicable (na), inductively coupled plasma mass spectrometry (ICP-MS), inductively coupled plasma optical emission spectrometry (ICP-OES). * denotes measures based on a range of means from seven study sites.

Reference	Com-pound	Method	Sample Material	Sample Size	Sampling Year	Country	Levels Detected (µg/g Dry Weight)	Levels Detected (µg/mL Wet Weight)	Mean Levels Detected (µg/g)	Median Levels Detected (µg/g or µg/mL)	Hedgehogs Analysed
**Arsenic**											
Vermeulen et al., 2009 [58]	Arsenic	ICP-MS	Blood	26	2005–2006	BE	na	0.1–155.5	na	0.2–17.8	Live, wild
D’Havé et al., 2006b [29]	Arsenic	ICP-MS	Fat	7	2002–2003	BE + NL	ND–0.13	na	0.08	na	Dead, roadkill + in care
Vermeulen et al., 2009 [58]	Arsenic	ICP-MS	Hair	26	2005–2006	BE	0.2–17.3	na	na	0.3–8.2	Live, wild
Rautio et al., 2010 [57]	Arsenic	ICP-OES	Hair	65	2004–2005	FI	ND–1.6	na	0.46	na	Dead, roadkill + starvation
D’Havé et al., 2006b [29]	Arsenic	ICP-MS	Hair	43	2002–2003	BE + NL	ND–2.33	na	0.69	na	Dead, roadkill + in care
D’Havé et al., 2005a [59]	Arsenic	ICP-OES	Hair	83	2002	BE	0.11–6.46 *	na	na	na	Live, wild
Rautio et al., 2010 [57]	Arsenic	ICP-OES	Kidney	64	2004–2005	FI	0.13–1.1	na	0.47	na	Dead, roadkill + starvation
D’Havé et al., 2006b [29]	Arsenic	ICP-MS	Kidney	44	2002–2003	BE + NL	ND–2.06	na	0.58	na	Dead, roadkill + in care
Rautio et al., 2010 [57]	Arsenic	ICP-OES	Liver	58	2004–2005	FI	0.26–1.06	na	0.45	na	Dead, roadkill + starvation
D’Havé et al., 2006b [29]	Arsenic	ICP-MS	Liver	43	2002–2003	BE + NL	ND–4.23	na	0.69	na	Dead, roadkill + in care
Jota Baptista et al., 2023 [63]	Arsenic	ICP-MS	Liver	41	2019–2021	PT	0–0.64	na	0.13	na	Dead, in care
D’Havé et al., 2006b [29]	Arsenic	ICP-MS	Muscle	44	2002–2003	BE + NL	ND–1.42	na	0.29	na	Dead, roadkill + in care
Vermeulen et al., 2009 [58]	Arsenic	ICP-MS	Spines	26	2005–2006	BE	0.2–23.6	na	na	0.4–6.3	Live, wild
Rautio et al., 2010 [57]	Arsenic	ICP-OES	Spines	63	2004–2005	FI	0.16–1.56	na	0.42	na	Dead, roadkill + starvation
D’Havé et al., 2006b [29]	Arsenic	ICP-MS	Spines	43	2002–2003	BE + NL	ND–5.66	na	1.24	na	Dead, roadkill + in care
D’Havé et al., 2005a [59]	Arsenic	ICP-OES	Spines	82	2002	BE	0.23–7.97 *	na	na	na	Live, wild
**Selenium**											
Rautio et al., 2010 [57]	Selenium	ICP-OES	Hair	65	2005–2006	FI	ND–1.02	na	0.18	na	Dead, roadkill + starvation
Rautio et al., 2010 [57]	Selenium	ICP-OES	Kidney	64	2004–2005	FI	2.05–11.36	na	4.63	na	Dead, roadkill + starvation
Rautio et al., 2010 [57]	Selenium	ICP-OES	Liver	58	2004–2005	FI	1.22–3.89	na	2.4	na	Dead, roadkill + starvation
Rautio et al., 2010 [57]	Selenium	ICP-OES	Spines	63	2004–2005	FI	0.05–1.65	na	0.69	na	Dead, roadkill + starvation

## Data Availability

All the data are available in the published studies used for this review.

## Supplementary Materials

The following supporting information can be downloaded at: https://www.mdpi.com/article/10.3390/ani14020232/s1. An overview of the prevalence and detection levels of metals in European hedgehogs. Abbreviations used: Belgium (BE), the Netherlands (NL), Finland (FI), Portugal (PT), Italy (I), not detected (ND), not applicable (na), inductively coupled plasma mass spectrometry (ICP-MS), inductively coupled plasma optical emission spectrometer (ICP-OES), atomic absorption spectrometry (AAS). * denotes measures based on a range of means from seven study sites. An overview of the prevalence and detection levels of metals in European hedgehogs. Abbreviations used: Belgium (BE), the Netherlands (NL), Finland (FI), Portugal (PT), Italy (I), not detected (ND), not applicable (na), inductively coupled plasma mass spectrometry (ICP-MS), inductively coupled plasma optical emission spectrometer (ICP-OES), atomic absorption spectrometry (AAS). * denotes measures based on a range of means from seven study sites.
