# Supplementary material for: A Review of the Occurrence of Metals and Xenobiotics in European Hedgehogs (Erinaceus europaeus)
Source: Animals (Basel). 2024 Jan 11;14(2):232. doi: 10.3390/ani14020232 (PMC10812797; doi:10.3390/ani14020232)
Supplement: Supplementary file 1 [file animals-14-00232-s001.zip › animals-2762891-supplementary.pdf]

Supplementary Materials. An overview of the prevalence and detection levels of metals in European hedgehogs. Abbreviations used: Belgium (BE), the Netherlands (NL), Finland (FI), Portugal (PT), Italy (I), not detected (ND), not applicable (na), inductively coupled plasma-mass spectrometry (ICP-MS), inductively coupled plasma-optical emission spectrometer (ICP-OES), atomic absorption spectrometry (AAS). * Denotes measures based on a range of means from seven study sites.

Reference	Compound	Method	Sample material	Sample size	Sampling year	Country	Levels detected (μg/g dry weight)	Levels detected (μg/mL wet weight)	Mean levels detected (μg/g)	Frequency of positives %	Hedgehogs analysed
**Ag**											
D'Havé et al. 2006b	**Ag**	ICP-MS	**Fat**	7	2002-2003	BE+NL	**ND-0.005**	na	**0.002**	na	Dead, road-killed + in care
D'Havé et al. 2006b	**Ag**	ICP-MS	**Hair**	43	2002-2003	BE+NL	**ND-0.19**	na	**0.02**	72	Dead, road-killed + in care
D'Havé et al. 2005a	**Ag**	ICP-OES	**Hair**	83	2002	BE	**0.004-0.317***	na	**na**	na	Live, wild hedgehogs
D'Havé et al. 2006b	**Ag**	ICP-MS	**Kidney**	44	2002-2003	BE+NL	**ND-0.62**	na	**0.04**	na	Dead, road-killed + in care
D'Havé et al. 2006b	**Ag**	ICP-MS	**Liver**	43	2002-2003	BE+NL	**ND-0.48**	na	**0.12**	na	Dead, road-killed + in care
D'Havé et al. 2006b	**Ag**	ICP-MS	**Muscle**	44	2002-2003	BE+NL	**ND-0.057**	na	**ND**	na	Dead, road-killed + in care
D'Havé et al. 2006b	**Ag**	ICP-MS	**Spines**	43	2002-2003	BE+NL	**ND-0.35**	na	**0.02**	81	Dead, road-killed + in care
D'Havé et al. 2005a	**Ag**	ICP-OES	**Spines**	82	2002	BE	**0.008-0.255***	na	**na**	na	Live, wild hedgehogs
**Al**											
Vermeulen et al. 2009	**Al**	ICP-MS	**Blood**	26	2005-2006	BE	**na**	0.5-17.7	**na**	100	Live, wild hedgehogs
D'Havé et al. 2006b	**Al**	ICP-MS	**Fat**	7	2002-2003	BE+NL	**ND-47**	na	**8**	na	Dead, road-killed + in care
D'Havé et al. 2006b	**Al**	ICP-MS	**Hair**	43	2002-2003	BE+NL	**1-331**	na	**42**	na	Dead, road-killed + in care
Vermeulen et al. 2009	**Al**	ICP-MS	**Hair**	26	2005-2006	BE	**1-6**	na	**na**	100	Live, wild hedgehogs
D'Havé et al. 2005a	**Al**	ICP-OES	**Hair**	83	2002	BE	**1.52-17.12***	na	**na**	na	Live, wild hedgehogs
D'Havé et al. 2006b	**Al**	ICP-MS	**Kidney**	44	2002-2003	BE+NL	**ND-63**	na	**15**	na	Dead, road-killed + in care
D'Havé et al. 2006b	**Al**	ICP-MS	**Liver**	43	2002-2003	BE+NL	**1-106**	na	**11**	na	Dead, road-killed + in care
D'Havé et al. 2006b	**Al**	ICP-MS	**Muscle**	44	2002-2003	BE+NL	**ND-129**	na	**18**	na	Dead, road-killed + in care

D'Havé et al. 2006b	**Al**	ICP-MS	**Spines**	43	2002-2003	BE+NL	**14-230**	na	**76**	na	Dead, road-killed + in care
Vermeulen et al. 2009	**Al**	ICP-MS	**Spines**	26	2005-2006	BE	**4-39**	na	**na**	100	Live, wild hedgehogs
D'Havé et al. 2005a	**Al**	ICP-OES	**Spines**	82	2002	BE	**10.55-62.93***	na	**na**	na	Live, wild hedgehogs
**Cd**											
Vermeulen et al. 2009	**Cd**	ICP-MS	**Blood**	26	2005-2006	BE	**na**	ND-0.42	**na**	50	Live, wild hedgehogs
D'Havé et al. 2006b	**Cd**	ICP-MS	**Fat**	7	2002-2003	BE+NL	**0.01-36**	na	**0.1**	na	Dead, road-killed + in care
Rautio et al. 2010	**Cd**	ICP-OES	**Hair**	65	2004-2005	FI	**ND-0.16**	na	**0.04**	100	Dead, road-killed + star-vation
D'Havé et al. 2006b	**Cd**	ICP-MS	**Hair**	43	2002-2003	BE+NL	**ND-23**	na	**0.83**	70	Dead, road-killed + in care
Vermeulen et al. 2009	**Cd**	ICP-MS	**Hair**	26	2005-2006	BE	**ND-1.3**	na	**na**	50	Live, wild hedgehogs
D'Havé et al. 2005a	**Cd**	ICP-OES	**Hair**	83	2002	BE	**0.01-1.08***	na	**na**	na	Live, wild hedgehogs
Rautio et al. 2010	**Cd**	ICP-OES	**Kidney**	64	2004-2005	FI	**0.71–21.35**	na	**5.74**	100	Dead, road-killed + star-vation
D'Havé et al. 2006b	**Cd**	ICP-MS	**Kidney**	44	2002-2003	BE+NL	**0.3-337**	na	**45.17**	na	Dead, road-killed + in care
Rautio et al. 2010	**Cd**	ICP-OES	**Liver**	58	2004-2005	FI	**0.26–7.68**	na	**1.81**	100	Dead, road-killed + star-vation
D'Havé et al. 2006b	**Cd**	ICP-MS	**Liver**	43	2002-2003	BE+NL	**0.1-124**	na	**13.39**	na	Dead, road-killed + in care
Jota Baptista et al. 2023	**Cd**	ICP-MS	**Liver**	41	2019-2021	PT	**ND-6.07**	na	**0.84**	na	Dead, in care
Alleva et al. 2006	**Cd**	AAS	**Liver**	unknown	1994-1995	I	**0.07-0.65**	na	**0.34**	na	Dead, road-killed
D'Havé et al. 2006b	**Cd**	ICP-MS	**Muscle**	44	2002-2003	BE+NL	**ND-10**	na	**0.64**	na	Dead, road-killed + in care
Rautio et al. 2010	**Cd**	ICP-OES	**Spines**	63	2004-2005	FI	**ND-0.08**	na	**0.04**	98	Dead, road-killed + star-vation
D'Havé et al. 2006b	**Cd**	ICP-MS	**Spines**	43	2002-2003	BE+NL	**ND-0.9**	na	**0.12**	93	Dead, road-killed + in care
Vermeulen et al. 2009	**Cd**	ICP-MS	**Spines**	26	2005-2006	BE	**ND-1.54**	na	**na**	50	Live, wild hedgehogs
D'Havé et al. 2005a	**Cd**	ICP-OES	**Spines**	82	2002	BE	**0.014-0.504***	na	**na**	na	Live, wild hedgehogs
**Co**											

D'Havé et al. 2006b	**Co**	ICP-MS	**Fat**	7	2002-2003	BE+NL	**ND-0.057**	na	**0.01**	100	Dead, road-killed + in care
D'Havé et al. 2006b	**Co**	ICP-MS	**Hair**	43	2002-2003	BE+NL	**ND-0.34**	na	**0.09**	98	Dead, road-killed + in care
D'Havé et al. 2005a	**Co**	ICP-OES	**Hair**	83	2002	BE	**0.014-0.055***	na	**na**	na	Live, wild hedgehogs
D'Havé et al. 2006b	**Co**	ICP-MS	**Kidney**	44	2002-2003	BE+NL	**0.05-13.66**	na	**0.99**	100	Dead, road-killed + in care
Jota Baptista et al. 2023	**Co**	ICP-MS	**Liver**	41	2019-2021	PT	**0.05-1.66**	na	**0.27**	na	Dead, in care
D'Havé et al. 2006b	**Co**	ICP-MS	**Liver**	43	2002-2003	BE+NL	**0.06-1.2**	na	**0.4**	100	Dead, road-killed + in care
D'Havé et al. 2006b	**Co**	ICP-MS	**Muscle**	44	2002-2003	BE+NL	**0.03-0.49**	na	**0.18**	100	Dead, road-killed + in care
D'Havé et al. 2006b	**Co**	ICP-MS	**Spines**	43	2002-2003	BE+NL	**ND-0.79**	na	**0.13**	100	Dead, road-killed + in care
D'Havé et al. 2005a	**Co**	ICP-OES	**Spines**	82	2002	BE	**0.020-0.058***	na	**na**	na	Live, wild hedgehogs
**Cr**											
Vermeulen et al. 2009	**Cr**	ICP-MS	**Blood**	26	2005-2006	BE	**na**	1.6-5.3	**na**	100	Live, wild hedgehogs
D'Havé et al. 2006b	**Cr**	ICP-MS	**Fat**	7	2002-2003	BE+NL	**0.3-1.5**	na	**0.8**	100	Dead, road-killed + in care
D'Havé et al. 2006b	**Cr**	ICP-MS	**Hair**	43	2002-2003	BE+NL	**1-30.9**	na	**5.4**	100	Dead, road-killed + in care
Vermeulen et al. 2009	**Cr**	ICP-MS	**Hair**	26	2005-2006	BE	**1.0-4.2**	na	**na**	100	Live, wild hedgehogs
D'Havé et al. 2005a	**Cr**	ICP-OES	**Hair**	83	2002	BE	**1.1-2.14***	na	**na**	na	Live, wild hedgehogs
D'Havé et al. 2006b	**Cr**	ICP-MS	**Kidney**	44	2002-2003	BE+NL	**0.3-7.4**	na	**3.4**	100	Dead, road-killed + in care
D'Havé et al. 2006b	**Cr**	ICP-MS	**Liver**	43	2002-2003	BE+NL	**1.1-5.7**	na	**3.9**	100	Dead, road-killed + in care
Jota Baptista et al. 2023	**Cr**	ICP-MS	**Liver**	41	2019-2021	PT	**0.02-0.7**	na	**0.12**	na	Dead, in care
Alleva et al. 2006	**Cr**	AAS	**Liver**	unknown	1994-1995	I	**ND**	na	**ND**	na	Dead, road-killed
D'Havé et al. 2006b	**Cr**	ICP-MS	**Muscle**	44	2002-2003	BE+NL	**1.6-8.4**	na	**4.6**	100	Dead, road-killed + in care
D'Havé et al. 2006b	**Cr**	ICP-MS	**Spines**	43	2002-2003	BE+NL	**0.8-9.4**	na	**4.3**	100	Dead, road-killed + in care

Vermeulen et al. 2009	**Cr**	ICP-MS	**Spines**	26	2005-2006	BE	**1.1-3.5**	na	**na**	100	Live, wild hedgehogs
D'Havé et al. 2005a	**Cr**	ICP-OES	**Spines**	82	2002	BE	**0.93-2.44***	na	**na**	na	Live, wild hedgehogs
**Cu**											
Vermeulen et al. 2009	**Cu**	ICP-MS	**Blood**	26	2005-2006	BE	**na**	2.5-14.4	**na**	100	Live, wild hedgehogs
D'Havé et al. 2006b	**Cu**	ICP-MS	**Fat**	7	2002-2003	BE+NL	**0.2-5.6**	na	**1.8**	100	Dead, road-killed + in care
Rautio et al. 2010	**Cu**	ICP-OES	**Hair**	65	2004-2005	FI	**13.39–55.98**	na	**24.38**	78	Dead, road-killed + starvation
D'Havé et al. 2006b	**Cu**	ICP-MS	**Hair**	43	2002-2003	BE+NL	**8-146**	na	**62**	100	Dead, road-killed + in care
Vermeulen et al. 2009	**Cu**	ICP-MS	**Hair**	26	2005-2006	BE	**15-33**	na	**na**	100	Live, wild hedgehogs
D'Havé et al. 2005a	**Cu**	ICP-OES	**Hair**	83	2002	BE	**17.17-23.27***	na	**na**	na	Live, wild hedgehogs
Rautio et al. 2010	**Cu**	ICP-OES	**Kidney**	64	2004-2005	FI	**8.84–38.07**	na	**17.17**	100	Dead, road-killed + starvation
D'Havé et al. 2006b	**Cu**	ICP-MS	**Kidney**	44	2002-2003	BE+NL	**3-85**	na	**39**	100	Dead, road-killed + in care
Rautio et al. 2010	**Cu**	ICP-OES	**Liver**	58	2004-2005	FI	**9.80–48.65**	na	**18.53**	100	Dead, road-killed + starvation
Jota Baptista et al. 2023	**Cu**	ICP-MS	**Liver**	41	2019-2021	PT	**12.11-102.91**	na	**35.66**	na	Dead, in care
D'Havé et al. 2006b	**Cu**	ICP-MS	**Liver**	43	2002-2003	BE+NL	**3-200**	na	**64**	100	Dead, road-killed + in care
D'Havé et al. 2006b	**Cu**	ICP-MS	**Muscle**	44	2002-2003	BE+NL	**2-40**	na	**22**	100	Dead, road-killed + in care
Rautio et al. 2010	**Cu**	ICP-OES	**Spines**	63	2004-2005	FI	**7.58–33.78**	na	**11.13**	100	Dead, road-killed + starvation
D'Havé et al. 2006b	**Cu**	ICP-MS	**Spines**	43	2002-2003	BE+NL	**4-49**	na	**21**	100	Dead, road-killed + in care
Vermeulen et al. 2009	**Cu**	ICP-MS	**Spines**	26	2005-2006	BE	**7.1-17.9**	na	**na**	100	Live, wild hedgehogs
D'Havé et al. 2005a	**Cu**	ICP-OES	**Spines**	82	2002	BE	**8.4-14.77***	na	**na**	na	Live, wild hedgehogs
**Fe**											
Vermeulen et al. 2009	**Fe**	ICP-MS	**Blood**	26	2005-2006	BE	**na**	761-2228	**na**	100	Live, wild hedgehogs
D'Havé et al. 2006b	**Fe**	ICP-MS	**Fat**	7	2002-2003	BE+NL	**8-240**	na	**81**	100	Dead, road-killed + in care

Rautio et al. 2010	**Fe**	ICP-OES	**Hair**	65	2004-2005	FI	**ND-827.46**	na	**25.3**	89	Dead, road-killed + starvation
D'Havé et al. 2006b	**Fe**	ICP-MS	**Hair**	43	2002-2003	BE+NL	**16-749**	na	**104**	100	Dead, road-killed + in care
Vermeulen et al. 2009	**Fe**	ICP-MS	**Hair**	26	2005-2006	BE	**ND-22**	na	**na**	100	Live, wild hedgehogs
D'Havé et al. 2005a	**Fe**	ICP-OES	**Hair**	83	2002	BE	**8.18-35.79***	na	**na**	na	Live, wild hedgehogs
Rautio et al. 2010	**Fe**	ICP-OES	**Kidney**	64	2004-2005	FI	**126.96–768.57**	na	**294.43**	100	Dead, road-killed + starvation
D'Havé et al. 2006b	**Fe**	ICP-MS	**Kidney**	44	2002-2003	BE+NL	**54-2336**	na	**879**	100	Dead, road-killed + in care
Rautio et al. 2010	**Fe**	ICP-OES	**Liver**	58	2004-2005	FI	**361.77–2849.76**	na	**1023.69**	100	Dead, road-killed + starvation
D'Havé et al. 2006b	**Fe**	ICP-MS	**Liver**	43	2002-2003	BE+NL	**199-7511**	na	**2339**	100	Dead, road-killed + in care
D'Havé et al. 2006b	**Fe**	ICP-MS	**Muscle**	44	2002-2003	BE+NL	**90-2393**	na	**669**	100	Dead, road-killed + in care
Rautio et al. 2010	**Fe**	ICP-OES	**Spines**	63	2004-2005	FI	**4.69–79.14**	na	**22.94**	100	Dead, road-killed + starvation
D'Havé et al. 2006b	**Fe**	ICP-MS	**Spines**	43	2002-2003	BE+NL	**35-2681**	na	**318**	100	Dead, road-killed + in care
Vermeulen et al. 2009	**Fe**	ICP-MS	**Spines**	26	2005-2006	BE	**11-90**	na	**na**	100	Live, wild hedgehogs
D'Havé et al. 2005a	**Fe**	ICP-OES	**Spines**	82	2002	BE	**34.37-160.31***	na	**na**	na	Live, wild hedgehogs
**Hg**											
Alleva et al. 2006	**Hg**	AAS	**Liver**	unknown	1994-1995	I	**0.19**	na	**0.06**	na	Dead, road-killed
**Mg**											
Rautio et al. 2010	**Mg**	ICP-OES	**Hair**	65	2004-2005	FI	**46.33–460.47**	na	**144.88**	100	Dead, road-killed + starvation
Rautio et al. 2010	**Mg**	ICP-OES	**Kidney**	64	2004-2005	FI	**360.58–938.21**	na	**600.36**	100	Dead, road-killed + starvation
Rautio et al. 2010	**Mg**	ICP-OES	**Liver**	58	2004-2005	FI	**519.22–1086.24**	na	**731.04**	100	Dead, road-killed + starvation
Rautio et al. 2010	**Mg**	ICP-OES	**Spines**	63	2004-2005	FI	**247.83–686.15**	na	**358.39**	100	Dead, road-killed + starvation
**Mn**											
Vermeulen et al. 2009	**Mn**	ICP-MS	**Blood**	26	2005-2006	BE	**na**	0.08-7.45	**na**	100	Live, wild hedgehogs

Rautio et al. 2010	**Mn**	ICP-OES	**Hair**	65	2004-2005	FI	**ND-31.11**	na	**2.21**	80	Dead, road-killed + starvation
Vermeulen et al. 2009	**Mn**	ICP-MS	**Hair**	26	2005-2006	BE	**ND-7.5**	na	**na**	100	Live, wild hedgehogs
D'Havé et al. 2005a	**Mn**	ICP-OES	**Hair**	83	2002	BE	**1.18-5.15***	na	**na**	na	Live, wild hedgehogs
Rautio et al. 2010	**Mn**	ICP-OES	**Kidney**	64	2004-2005	FI	**0.90–5.44**	na	**2.38**	100	Dead, road-killed + starvation
Rautio et al. 2010	**Mn**	ICP-OES	**Liver**	58	2004-2005	FI	**1.69–19.18**	na	**6.33**	100	Dead, road-killed + starvation
Rautio et al. 2010	**Mn**	ICP-OES	**Spines**	63	2004-2005	FI	**ND-9.48**	na	**1.85**	79	Dead, road-killed + starvation
Vermeulen et al. 2009	**Mn**	ICP-MS	**Spines**	26	2005-2006	BE	**ND-8.6**	na	**na**	100	Live, wild hedgehogs
D'Havé et al. 2005a	**Mn**	ICP-OES	**Spines**	82	2002	BE	**1.67-13.61***	na	**na**	na	Live, wild hedgehogs
**Mo**											
Rautio et al. 2010	**Mo**	ICP-OES	**Hair**	65	2004-2005	FI	**ND**	na	**ND**	0	Dead, road-killed + starvation
Rautio et al. 2010	**Mo**	ICP-OES	**Kidney**	64	2004-2005	FI	**0.29–1.96**	na	**0.78**	100	Dead, road-killed + starvation
Rautio et al. 2010	**Mo**	ICP-OES	**Liver**	58	2004-2005	FI	**1.47–5.12**	na	**2.55**	100	Dead, road-killed + starvation
Rautio et al. 2010	**Mo**	ICP-OES	**Spines**	63	2004-2005	FI	**ND**	na	**ND**	0	Dead, road-killed + starvation
**Ni**											
Vermeulen et al. 2009	**Ni**	ICP-MS	**Blood**	26	2005-2006	BE	**na**	ND-0.34	**na**	31	Live, wild hedgehogs
D'Havé et al. 2006b	**Ni**	ICP-MS	**Fat**	7	2002-2003	BE+NL	**0.02-0.3**	na	**0.11**	na	Dead, road-killed + in care
Rautio et al. 2010	**Ni**	ICP-OES	**Hair**	65	2004-2005	FI	**ND-8.82**	na	**0,49**	91	Dead, road-killed + starvation
D'Havé et al. 2006b	**Ni**	ICP-MS	**Hair**	43	2002-2003	BE+NL	**ND-7.63**	na	**0.73**	95	Dead, road-killed + in care
Vermeulen et al. 2009	**Ni**	ICP-MS	**Hair**	26	2005-2006	BE	**ND-35**	na	**na**	31	Live, wild hedgehogs
D'Havé et al. 2005a	**Ni**	ICP-OES	**Hair**	83	2002	BE	**0.19-0.57***	na	**na**	na	Live, wild hedgehogs
Rautio et al. 2010	**Ni**	ICP-OES	**Kidney**	64	2004-2005	FI	**ND-2.11**	na	**0.17**	75	Dead, road-killed + starvation

D'Havé et al. 2006b	**Ni**	ICP-MS	**Kidney**	44	2002-2003	BE+NL	**0.03-2.38**	na	**0.3**	na	Dead, road-killed + in care
Rautio et al. 2010	**Ni**	ICP-OES	**Liver**	58	2004-2005	FI	**ND-0.83**	na	**0.07**	45	Dead, road-killed + star-vation
D'Havé et al. 2006b	**Ni**	ICP-MS	**Liver**	43	2002-2003	BE+NL	**0.01-0.77**	na	**0.2**	na	Dead, road-killed + in care
D'Havé et al. 2006b	**Ni**	ICP-MS	**Muscle**	44	2002-2003	BE+NL	**ND-1.26**	na	**0.28**	na	Dead, road-killed + in care
Rautio et al. 2010	**Ni**	ICP-OES	**Spines**	63	2004-2005	FI	**ND-0.69**	na	**0.14**	97	Dead, road-killed + star-vation
D'Havé et al. 2006b	**Ni**	ICP-MS	**Spines**	43	2002-2003	BE+NL	**ND-3.34**	na	**0.61**	98	Dead, road-killed + in care
Vermeulen et al. 2009	**Ni**	ICP-MS	**Spines**	26	2005-2006	BE	**ND-0.19**	na	**na**	31	Live, wild hedgehogs
D'Havé et al. 2005a	**Ni**	ICP-OES	**Spines**	82	2002	BE	**0.16-0.8***	na	**na**	na	Live, wild hedgehogs
Pb											
Vermeulen et al. 2009	**Pb**	ICP-MS	**Blood**	26	2005-2006	BE	**na**	4.9-937	**na**	100	Live, wild hedgehogs
Rautio et al. 2010	**Pb**	ICP-OES	**Hair**	65	2004-2005	FI	**ND-7.1**	na	**0.98**	78	Dead, road-killed + star-vation
D'Havé et al. 2006b	**Pb**	ICP-MS	**Hair**	43	2002-2003	BE+NL	**0.2-15.4**	na	**2.6**	100	Dead, road-killed + in care
Vermeulen et al. 2009	**Pb**	ICP-MS	**Hair**	26	2005-2006	BE	**0.1-14.1**	na	**na**	100	Live, wild hedgehogs
D'Havé et al. 2005a	**Pb**	ICP-OES	**Hair**	83	2002	BE	**0.28-11.7***	na	**na**	na	Live, wild hedgehogs
Rautio et al. 2010	**Pb**	ICP-OES	**Kidney**	64	2004-2005	FI	**ND-4.5**	na	**0.95**	97	Dead, road-killed + star-vation
D'Havé et al. 2006b	**Pb**	ICP-MS	**Kidney**	44	2002-2003	BE+NL	**0.2-18.3**	na	**5.2**	100	Dead, road-killed + in care
Rautio et al. 2010	**Pb**	ICP-OES	**Liver**	58	2004-2005	FI	**ND-3.90**	na	**1.03**	97	Dead, road-killed + star-vation
D'Havé et al. 2006b	**Pb**	ICP-MS	**Liver**	43	2002-2003	BE+NL	**0.7-71**	na	**10.9**	100	Dead, road-killed + in care
Jota Baptista et al. 2023	**Pb**	ICP-MS	**Liver**	41	2019-2021	PT	**0.09-4.46**	na	**0.54**	na	Dead, in care
Alleva et al. 2006	**Pb**	AAS	**Liver**	unknown	1994-1995	I	**0.62-1.28**	na	**0.79**	na	Dead, road-killed
D'Havé et al. 2006b	**Pb**	ICP-MS	**Muscle**	44	2002-2003	BE+NL	**0.1-31.5**	na	**1,8**	100	Dead, road-killed + in care

Rautio et al. 2010	**Pb**	ICP-OES	**Spines**	63	2004-2005	FI	**ND-7.02**	na	**0.54**	98	Dead, road-killed + star-vation
D'Havé et al. 2006b	**Pb**	ICP-MS	**Spines**	43	2002-2003	BE+NL	**0.5-13.7**	na	**3.8**	100	Dead, road-killed + in care
Vermeulen et al. 2009	**Pb**	ICP-MS	**Spines**	26	2005-2006	BE	**0.2-17.2**	na	**na**	100	Live, wild hedgehogs
D'Havé et al. 2005a	**Pb**	ICP-OES	**Spines**	82	2002	BE	**0.58-11.79***	na	**na**	na	Live, wild hedgehogs
**Zn**											
Vermeulen et al. 2009	**Zn**	ICP-MS	**Blood**	26	2005-2006	BE	**na**	29-209	**na**	100	Live, wild hedgehogs
D'Havé et al. 2006b	**Zn**	ICP-MS	**Fat**	7	2002-2003	BE+NL	**0.1-1.4**	na	**0.6**	100	Dead, road-killed + in care
D'Havé et al. 2006b	**Zn**	ICP-MS	**Fat**	7	2002-2003	BE+NL	**3-43**	na	**21**	100	Dead, road-killed + in care
Rautio et al. 2010	**Zn**	ICP-OES	**Hair**	65	2004-2005	FI	**134.28–217.50**	na	**167.85**	100	Dead, road-killed + star-vation
D'Havé et al. 2006b	**Zn**	ICP-MS	**Hair**	43	2002-2003	BE+NL	**49-426**	na	**201**	100	Dead, road-killed + in care
Vermeulen et al. 2009	**Zn**	ICP-MS	**Hair**	26	2005-2006	BE	**100-267**	na	**na**	100	Live, wild hedgehogs
D'Havé et al. 2005a	**Zn**	ICP-OES	**Hair**	83	2002	BE	**99.23-147.57***	na	**na**	na	Live, wild hedgehogs
Rautio et al. 2010	**Zn**	ICP-OES	**Kidney**	64	2004-2005	FI	**52.15–161.57**	na	**103.22**	100	Dead, road-killed + star-vation
D'Havé et al. 2006b	**Zn**	ICP-MS	**Kidney**	44	2002-2003	BE+NL	**27-441**	na	**135**	100	Dead, road-killed + in care
Rautio et al. 2010	**Zn**	ICP-OES	**Liver**	58	2004-2005	FI	**79.96–586.88**	na	**228.97**	100	Dead, road-killed + star-vation
D'Havé et al. 2006b	**Zn**	ICP-MS	**Liver**	43	2002-2003	BE+NL	**50-747**	na	**371**	100	Dead, road-killed + in care
D'Havé et al. 2006b	**Zn**	ICP-MS	**Muscle**	44	2002-2003	BE+NL	**48-299**	na	**177**	100	Dead, road-killed + in care
Rautio et al. 2010	**Zn**	ICP-OES	**Spines**	63	2004-2005	FI	**80.59–108.90**	na	**90.93**	100	Dead, road-killed + star-vation
D'Havé et al. 2006b	**Zn**	ICP-MS	**Spines**	43	2002-2003	BE+NL	**36-353**	na	**153**	100	Dead, road-killed + in care
Vermeulen et al. 2009	**Zn**	ICP-MS	**Spines**	26	2005-2006	BE	**56-135**	na	**na**	100	Live, wild hedgehogs
D'Havé et al. 2005a	**Zn**	ICP-OES	**Spines**	82	2002	BE	**62.98-94.18***	na	**na**	na	Live, wild hedgehogs
